# Reality Meets Belief: A Mixed Methods Study on Character Strengths and Well-Being of Hospital Physicians

**DOI:** 10.3389/fpsyg.2021.547773

**Published:** 2021-06-10

**Authors:** Timo Kachel, Alexandra Huber, Cornelia Strecker, Thomas Höge, Stefan Höfer

**Affiliations:** ^1^Institute of Psychology, University of Innsbruck, Innsbruck, Austria; ^2^Department of Medical Psychology, Medical University Innsbruck, Innsbruck, Austria

**Keywords:** mixed-methods, burnout, work engagement, well-being, character strengths, hospital physicians, tertiary split

## Abstract

Positive psychology deals with factors that make life most worth living and focuses on enhancing individual potentials. Particularly, character strengths can positively contribute to well-being and work-related health, bearing a promising potential for professions, such as physicians, who are at risk for burnout or mental illnesses. This study aims to identify beneficial character strengths by examining the quantitative and qualitative data. In a cross-sectional multi-method study, 218 hospital physicians completed an online survey assessing their character strengths and their general and work-related well-being, comprising thriving, work engagement, and burnout dimensions (outcome variables). Quantitative data were analyzed for the total sample and by tertiary split. Additionally, interview-gathered opinions of four resident physicians and four medical specialist educators were collected to expand the perspective on which character strengths might be beneficial for the well-being of the resident physicians. The highest significant correlations between character strengths and outcome variables were found for *hope* and thriving (*r* = 0.67), *zest*, and work engagement (*r* = 0.67) as well as emotional exhaustion (*r* = −0.47), *perseverance/leadership* and depersonalization (*r* = −0.27), *bravery*, and reduced personal accomplishment (*r* = −0.39). Tertiary splits revealed that some correlations were not consistent across the entire scale continuum, for example, *creativity* was only significantly correlated with comparatively high levels of thriving (*r* = 0.28) or *forgiveness* with comparatively high levels of depersonalization (*r* = −0.34). *Humility*, s*ocial intelligence*, and *teamwork* showed predominantly low correlations with all outcome variables (*r* = −0.17 − 0.34), although *humility* was stated by all interviewed medical specialist educators to be the most relevant for the well-being at work, and the latter two by three resident physicians, respectively. Different perspectives resulting from quantitative and qualitative data in terms of beneficial character strengths for work-related well-being may be driven by different work experiences, professional understandings, generational beliefs, or social expectations. Some significant correlations between character strengths and well-being outcomes varied depending on low, medium, or high outcomes. This raises questions about suitable work-related well-being interventions, as simple single intervention approaches (one intervention fits all) may not work for the respective outcome levels. These new findings warrant further research on how to foster the well-being of resident physicians at work.

## Introduction

Already in ancient times, concepts like hedonism, happiness, and “the good life” were ascribed a special meaning. This view has been expanded over the centuries, adding concepts and theories. In the last few decades, psychological research has paid particular attention to this topic resulting in various approaches that deal with well-being in relation to emotional, subjective, and dedicated areas of life. Most recently, efforts have been made to unite these theories to one concept of “thriving” (Su et al., 2014). Factors being beneficial for well-being or rather thriving and individual potentials are investigated by positive psychology (Seligman and Csikszentmihalyi, 2000). This discipline has existed since the beginning of the year 2000 and deals with positively valued personal characteristics, namely character strengths, which enable growth, flourishing, and moral excellence further gaining positive benefits by utilizing them (Seligman, 2002). Studies have shown that explicitly focusing on well-being, particularly among the medical staff in the hospital, compared to burnout, is a rather neglected research topic. Burnout in medical staff is higher compared to the general population (Dyrbye et al., 2014). This might be due to environmental and personal factors. Though alternation of environmental (work-related) factors in the hospital context (e.g., demands, workload, resources, organizational values, social support, and work-life integration) can usually only be achieved by extensive changes to the existing systems, it is more expedient to focus on personal/individual factors (e.g., traits, skills, stress-coping behavior, and internal conflicts) in order to promote the well-being and prevent illness at the best. This study utilizes a mixed-methods approach to address three aims constituting the relations between individual character strengths of hospital physicians and work-related well-being (thriving, work engagement, and burnout), analyzing the associations of individual character strengths with respect to the entire spectrum of work-related well-being outcomes by tertiary split (Gelman and Park, 2009), and adding qualitative data by identifying the most relevant character strengths for well-being in the hospital *via* interview-gathered opinions of resident physicians and medical specialist educators.

### Today's Situation in Hospitals

The current social role of hospitals as institutions, the perceptions of organizational processes, and the exposure to employees have changed within the last decades (Cockerham, 2014). Previously recognized personal and environmental demands at work and job strains (e.g., lack of autonomy, inadequate resources, organizational stressors, workload, and adverse working hours; Lee et al., 2013; Angerer and Weigl, 2015) are pressuring physicians as well as new elementary challenges, for example, regarding the delivery of healthcare or organization (Arnetz, 2001), financial pressure (Bazzoli et al., 2007), a worsened psychosocial work environment due to increasing workplace violence emanating from patients and their relatives toward health care professionals (Hahn et al., 2008), new health information technology for patients (Goldzweig et al., 2009), or the expectations of the public and patients (Mechanic, 2003). Negative consequences due to these grievances are confirmed by current studies. According to Dyrbye et al. (2014), up to 60% of resident physicians experienced symptoms of burnout, and depending on each specialty, 27–75% were affected by them (Ishak et al., 2009), leading to increased medical errors (West et al., 2006). In Austria and Germany, researchers have found a similar prevalence of burnout (up to 50%) and depression (10%) in physicians (Weigl et al., 2012; Wurm et al., 2016). Kachel et al. (2020) described the development of the beginning of burnout-related symptoms during medical school. Furthermore, Klein et al. (2010) stated a significant link between the perceived stress of the physicians at work and their quality of care directly affecting the patients. In light of this situation, physicians are not only exposed to these circumstances but also responsible for changes either toward or away from improved health care settings (Porter and Teisberg, 2007).

### Character Strengths

Character strengths are conceptualized as positive traits or components of the “good character,” therefore morally valued and nurtured by individuals or institutions (Peterson and Seligman, 2004). Their definition implies some degree of stability. However, the authors assume that character strengths are not “set in stone” and the environment can also shape them (Peterson and Seligman, 2004; Park and Peterson, 2009). Traits, as known by the Big Five personality model from Allport (1937), differ regarding their descriptive perspective on individual differences in contrast to the normative value of character strengths. In the “Values-in-Action” (VIA) classification of character strengths (Peterson and Seligman, 2004), 24 character strengths are allocated to the following six theoretically constructed virtues: (1) wisdom and knowledge (*creativity, curiosity, judgment, love of learning*, and *perspective*); (2) courage (*bravery, honesty, perseverance*, and *zest*); (3) humanity (*kindness, love*, and *social intelligence*); (4) justice (*fairness, leadership*, and *teamwork*); (5) temperance (*forgiveness, modesty, prudence*, and *self-regulation*), and (6) transcendence (*appreciation of beauty and excellence, gratitude, hope, humor*, and *spirituality*). Some character strengths are phasic (their use is only relevant in specific situations, e.g., *bravery*) whereas others can be used constantly in a broad variety of situations being tonic (e.g., *kindness, humor*; Harzer and Ruch, 2013). Character strengths were also characterized as “neglected but critically important resources for organizations” (Peterson and Park, 2006). Pursuant to Seligman (2002), it is relevant to apply these character strengths in important life domains to obtain satisfaction and well-being.

### Thriving and Character Strengths

The concept of thriving denotes the state of positive functioning at its fullest range—mentally, physically, and socially (Su et al., 2014). Thriving includes the core dimensions of well-being and corresponding constructs from prominent positive psychology theories (Su et al., 2014), that is, Diener's subjective well-being, Ryff's psychological well-being, self-determination of Ryan and Deci, PERMA model of Seligman, and the optimism of Scheier and Carver. Therefore, this comprehensive construct enables a holistic perspective of positive functioning, as thriving in life is not only defined by feelings of happiness, or a sense of autonomy, or having positive relationships, but it is a collection of all these facets. All humans are situated somewhere on the continuum from low to high levels of thriving. Keyes (2002) emphasized the importance of this view by the results of one of his studies where the risk of mental illness was higher when people reported lower levels of well-being. Whereas, among people with higher well-being levels, the risk of being affected by a major depressive episode was almost six times lower. There have been some analyses showing that the level of character strength in the context of the medical profession can be associated with aspects of thriving. For example, a study conducted by Hausler et al. (2017b) found strong positive associations (descending order) between the character strengths, *hope, zest, gratitude, love*, and *curiosity* with psychological and subjective well-being in a sample of medical students. Furthermore, according to the results provided by Huber et al. (2019), the character strengths, such as *fairness, honesty*, and *kindness* showed relations with subjective well-being, whereas *judgment* and *kindness* had negative associations with reduced personal accomplishment. Besides these results, strengths used in general had been positively associated with vitality, self-esteem, and positive affect (Huber et al., 2017). Moreover, some studies indicated relationship between the applicability of character strengths and mental health as well as thriving (Hausler et al., 2017c; Strecker et al., 2019), work engagement and burnout (Huber et al., 2019), and the socio-moral organizational climate in the hospital context (Höge et al., 2019).

### Work Engagement and Burnout

In particular, work engagement and burnout can be seen as important parts of work-related well-being. Work engagement is defined as a fulfilling, positive, work-related ambitious state of mind and characterized by three components, such as vigor, dedication, and absorption (Schaufeli et al., 2002). Vigor is defined as a mental resilience having high levels of energy while working, dedication is characterized by a strong involvement in work tasks, and absorption means being fully concentrated on one's work, whereby time passes quickly. Positive psychology was inspiring for this concept (Schaufeli et al., 2002), which is why it focuses on health- and personality-promoting effects of work, while looking at work-related well-being. Job burnout could be considered as the negative counterpart of work engagement with the following three dimensions: emotional exhaustion (reduced emotional/internal resources, feelings of having nothing more to give to the job), depersonalization (trying to distance oneself from the job, increasing cynicism about the value of work, actively starting to ignore positive aspects of the job), and reduced personal accomplishment (feelings of less effectiveness in the job, performance decreases; Maslach et al., 2001). According to the Job-Demands-Resources model by Bakker and Demerouti (2007), burnout shows up when resources are no longer sufficient to cope with stressors at work. So far it has been shown that the underuse as well as the overuse of character strengths has significant relations to lower well-being and mental illness in general (Freidlin et al., 2017; Littman-Ovadia and Freidlin, 2019). Although, previous studies made an important contribution to the illustration of the relationships between character strengths, work engagement, and burnout (Hausler et al., 2017c; Huber et al., 2019), there are still ambiguities whether specific character strengths are relevant at different levels of work-related well-being in the context of medical professionals.

### Physicians' Well-Being and Burnout

For better understanding of the links between the well-being and burnout among physicians, it is important to briefly address relevant conceptual framework models. Stewart et al. (2019) provided a compilation of the most relevant models regarding this issue. The *PERMA*-model (acronym for positive emotions, engagement, relationship, meaning, and achievement; Seligman, 2011) integrates five concepts from the hedonic and eudemonic tradition of well-being research. Recently this model has been expanded by a sixth element, called “health” (The Wellbeing Lab, 2021). The PERMA-model could be employed in hospitals and medical schools two-fold (Slavin et al., 2012); it can be used to help individuals develop resources (e.g., emotional and cognitive tools) and to provide institutions with a “blueprint” of interventions that facilitate cultural change fostering the well-being of students and physicians. The *coping reserve* model, developed by Dunn et al. (2008), characterizes resilience as a dynamic process. It describes a tank (coping reserve) that is filled by factors like psychosocial support, social and health activities, or mentorship and is drained by factors like stress, time and energy demands, or internal conflicts. The bottom line filling is determined by the temperament and personality of an individual. If depleting factors are equal to or fall below replenishing factors, resilience can be achieved. On the institutional level, this model can help make efforts to reduce draining factors and promote filling factors. Furthermore, it helps physicians to understand the contribution of self-care and burnout prevention. Shanafelt and Noseworthy (2017) presented a diametral model that describes “drivers” of the burnout and engagement continuum. These drivers are divided into seven main dimensions, such as workload and job demands, control and flexibility, work-life integration, social support and community at work, organizational culture and values, efficiency and resources, and meaning in work. The authors provided a comprehensive presentation of examples of contributing factors on an individual, work unit, organizational, and national level for each driver. The Stanford WellMD model of professional fulfillment (Bohman et al., 2017) describes drivers in three different categories, such as personal resilience, efficiency of practice, and culture of wellness. The first domain includes factors like healthy nutrition, sleep hygiene, or regular exercise and lies in the individual's responsibility to be fulfilled. The latter two are in the responsibility of the organization, including factors like quality of mentoring or leadership or adjustable timetables (culture of wellness) and staffing models or electronic health records (efficiency of practice).

## Aims

Several studies have highlighted the association of character strengths with different aspects of thriving and other health-related outcomes in the field of the medical profession in principle. However, there is a lack of studies investigating in detail about the correlations between character strengths and the entire well-being spectrum among clinicians. To this end, using trichotomization of the outcome variables (Gelman and Park, 2009), character strengths will be examined more closely in terms of their relationships to different aspects of well-being (thriving, work engagement, and burnout dimensions) along their continuum. Specifically, this analysis will for the first time illustrate the strength of the relationship between individual character strengths and health-related outcomes in dependence of different outcome-levels. Qualitative data concerning the perceived importance of individual character strengths for well-being in the hospital context will highlight the similarities or discrepancies among the actual impact of character strengths on the well-being (quantitative data) bringing new insights regarding future management strategies.

Therefore, this study aimed to investigate the following:

(1) Whether there are specific character strengths correlating substantially higher with the outcome variables of the well-being of the resident physicians (thriving, work engagement, and burnout dimensions) than others. This is of particular importance as the results could be used to develop intervention programs that specifically focus on promoting these character strengths in order to help physicians deal with the challenges of the hospitals, which may affect their well-being. In addition, the results may reveal possible clear or tending relationships across various outcome variables.

(2) Whether correlations between character strengths and well-being outcome variables (thriving, work engagement, and burnout dimensions) are significant across the entire continuum of the respective measurement scale or if there are differences when split up into low, medium, and high outcome groups. The intention here is not to “diagnose” or differentiate between “low or high burnout.” Rather than answering this question, one can detect significant patterns in the data that would have been undiscovered when solely looking at the total scale means, thus providing a deeper understanding of whether character strengths are of varying importance for different graduations of work-related well-being in the context of medical staff. Furthermore, understanding these relations can also be used to develop targeted intervention programs for certain outcome levels.

(3) Whether a sub-sample of interviewed resident physicians in training and their medical specialist educators share beliefs on which character strengths are most important for the well-being of the resident physicians in the hospital, and how these beliefs complement quantitative empirical data as to the relationship of specific character strengths and the outcome variables of well-being. Their answers potentially enhance the perspective on perceived relations between character strengths and well-being among resident physicians from a “social constructivist” and more subjective perspective.

## Methods

### Participants and Procedure

With institutional approval from the review board, a maximum of 700 resident physicians were invited *via* e-mail to complete an anonymous online questionnaire. Data were collected between May 2015 and December 2016 at the main and secondary Austrian hospitals. Out of 274 responding participants (39.1% return rate), 218 complete data sets (79.6%) were available for the underlying analyses. Of all questionnaire attendees, 79.4% [*n* = 173; *M*_age_ = 31.8 years (*SD* ± 5.0; range 24–50)] were resident physicians and 20.6% [*n* = 45; *M*_age_ = 45.7 years (*SD* ± 10.0; range 27–64)] were medical specialist educators. Resident physicians and medical specialist educators worked in 16 different medical specialties. The proportion of female participants in total was 61.5%. Of all attendees, 76.6% were in a relationship or married, and 67% lived with their partner. About 72.9% were Austrians followed by 12.8% Germans and 11% Italians. Among the attending physicians, 31.6% had at least one child. About one quarter of the participating physicians (21.1%) had a contract with more than 40 working hours per week, but in fact 79.8% worked more than 40 h per week. In total, 69.9% of the physicians had a permanent contract lasting for the whole duration of their medical specialist training. Additionally, interviews with four resident physicians (*M*_age_ = 33 years, 50% female) and four medical specialist educators (*M*_age_ = 51 years, 25% female) at the same main hospital facility were conducted during February and March 2017.

### Quantitative Measures

#### Character Strengths

The “Values in Action—Inventory of Strengths” (VIA-IS; Peterson et al., 2005) was used to measure individual character strengths. The German short version (Höfer et al., 2019) consists of 120 items, capturing 24 character strengths by five items each. They are rated on a five-point scale from 1 = *strongly disagree* to 5 = *strongly agree*. Item examples, include “Being able to come up with new and different ideas is one of my strong points” (creativity), “I am very disciplined” (self-regulation) or “I like being nice to others” (kindness). In this sample, the internal consistency ranged between α = 0.63 (teamwork and perspective) and α = 0.91 (spirituality).

#### Thriving

The German version of the “Comprehensive Inventory of Thriving” (CIT; Hausler et al., 2017a) was used to measure the well-being in general that is, thriving. All components of thriving are measured by the following 18 subscales: life satisfaction, positive and negative feelings; support, community, trust, respect, loneliness, belonging (together building the scale “relationship”), skills, learning, accomplishment, self-efficacy, self-worth (together building the scale, “mastery”), engagement, autonomy, meaning, and optimism. The 54 items are rated on a five-point scale ranging from 1 = *strongly disagree* to 5 = *strongly agree*. Item examples, include “I use my skills a lot in my everyday life” (mastery) or “People respect me” (relationship). Hausler et al. (2017a) validated the German version, demonstrating its reliability, and validity. Cronbach's alpha in this sample was α = 0.95.

#### Work Engagement

The German short version of the “Utrecht Work Engagement Scale” (UWES; Schaufeli and Bakker, 2003; Schaufeli et al., 2006) was utilized to measure work engagement unidimensionally (Schaufeli et al., 2006). Work engagement is defined as a fulfilling work-related positive state of mind that is characterized by absorption, dedication, and vigor. Schaufeli et al. (2006) recommended expressing these dimensions by means of a total score in the short version to avoid problems with multicollinearity in regression analyses. Moreover, the three-factor structure was reported to be invariant in explorative analyses (Sonnentag, 2003) as well as across different samples (Schaufeli et al., 2002); therefore we did not report subscale scores. The nine items are rated on a seven-point scale ranging from 0 = *never* to 6 = *always*. Item examples are as follows: “At my work, I feel bursting with energy” or “When I get up in the morning, I feel like going to work.” Cronbach's alpha in this sample was α = 0.94.

#### Burnout

Burnout dimensions were measured with the German version of the “Maslach-Burnout-Inventory” (MBI-D; Büssing and Perrar, 1992), including emotional exhaustion, depersonalization, and reduced personal accomplishment. They are measured with 21 items and rated on a six-point scale ranging from 0 = *never* to 5 = *very often*. Item examples are as follows: “I feel emotionally exhausted through my work” (emotional exhaustion), “I feel emotionally drained from my work” (depersonalization), or “I feel very energetic” (reduced personal accomplishment). Cronbach's alpha in this sample ranged between α = 0.71 (depersonalization) and α = 0.91 (emotional exhaustion).

#### Content Analysis

Exemplarily, four resident physicians and four medical specialist educators, one of each belonging to the same department in the main hospital, were asked about their opinions regarding the perceived importance of character strengths for the well-being of resident physicians at work. This ranking question used here was part of a comprehensive qualitative methods section of the overarching project, which included several interview questions as well as field observations (Kachel et al., 2019). With regard to the interviews, the question presented here was only one of the relevances for the third hypothesis of this article, which is only why this question was used for further analyses. The four departments were selected in accordance with the highest number of participants in the quantitative sample coming from the respective specialist areas, and based on the representation of the largest specialist areas in medicine in general. All interviewees gave written informed consent and all interviews were audio-recorded. The interviewees were shown a list of all 24 character strengths from the VIA-classification (Peterson and Seligman, 2004), including a brief explanation. The question used from the qualitative study (Kachel et al., 2019) used in this analysis was as follows:

*In your opinion, which out of these 24 character strengths are the five most important a resident physician in the hospital needs to feel well*?

#### Data Analysis

In the first step, Pearson correlation analyses were applied to the quantitative data (SPSS version 26, IBM Corp., Armonk, NY, United States; IBM Corp., 2019) in order to identify possible relations between measured character strengths, thriving, work engagement, and burnout dimensions. In the second step, the tertiary split was carried out by building three equally sized groups for each outcome variable (*n* = 72|73).The group assignment followed the sample specific distribution of scale scores resulting in a low-level, mid-level, and high-level group. This tertiary split was conducted in order to identify specific patterns in the strength of relationships between the 24 character strengths and different outcome levels, respectively (Gelman and Park, 2009). Due to the plurality of predictors (*n* = 24) and partial multicollinearity, the Benjamini-Hochberg procedure was applied (Benjamini and Hochberg, 1995). The recordings of the structured interviews were transcribed literally and the mentioned character strengths counted.

## Results

### Descriptive Statistics

Table 1 presents the means and SD of the 24 character strengths of the whole sample. The highest mean level was found for the character strength, *honesty* (*M* = 4.23, *SD* = 0.43), the lowest mean level for *spirituality* (*M* = 2.37, *SD* = 0.99). Table 2 shows the means, SD, and minimum/maximum scores of the general and work-related outcome measures for the whole sample as well as by tertiary split. The mean of thriving for the whole sample ranged in the upper third of the entire scale spectrum ranged from 1 to 5 (*M* = 3.85, *SD* = 0.43); depersonalization as a component of burnout had the lowest mean score in total (*M* = 1.48, *SD* = 0.78, range 0–5).

**Table 1:** Descriptive statistics of the 24 character strengths (in alphabetical order).

**Character strength**	***N***	**α**	***M***	***SD***	**Min**.	**Max**.	**Skew**	**Kurtosis**
Appreciation of beauty	218	0.73	3.50	0.66	1.60	5.00	−0.09	−0.45
Bravery	218	0.73	3.42	0.63	2.20	5.00	0.02	−0.51
Creativity	218	0.85	3.44	0.70	1.00	5.00	−0.32	0.47
Curiosity	218	0.73	3.87	0.55	2.20	5.00	−0.38	−0.11
**Fairness**	218	0.75	4.04	0.56	2.00	5.00	−0.66	0.63
Forgiveness	218	0.66	3.44	0.63	1.80	5.00	0.01	−0.15
Gratitude	218	0.77	3.54	0.62	1.80	5.00	0.03	−0.21
**Honesty**	218	0.64	4.23	0.43	2.80	5.00	−0.35	−0.01
Hope	218	0.71	3.69	0.62	2.00	5.00	−0.35	−0.21
Humility	218	0.68	3.33	0.63	1.40	4.80	−0.14	−0.06
Humor	218	0.83	3.69	0.68	2.00	5.00	−0.08	−0.35
**Judgment**	218	0.72	4.04	0.50	2.80	5.00	−0.18	−0.33
**Kindness**	218	0.75	4.12	0.52	2.80	5.00	−0.27	−0.18
Leadership	218	0.68	3.70	0.53	2.00	5.00	0.05	0.05
**Love**	218	0.80	4.04	0.69	1.60	5.00	−0.87	0.88
Love of learning	218	0.74	3.65	0.67	1.60	5.00	−0.20	−0.27
Perseverance	218	0.77	3.93	0.60	2.00	5.00	−0.65	0.44
Perspective	218	0.63	3.39	0.52	2.00	5.00	−0.04	0.18
Prudence	218	0.70	3.51	0.60	1.80	5.80	−0.23	−0.20
Self-regulation	218	0.65	3.15	0.70	1.20	5.80	−0.18	−0.09
Social intelligence	218	0.72	3.90	0.53	2.40	5.00	−0.34	−0.06
Spirituality	218	0.91	2.37	0.99	1.00	5.00	0.56	−0.40
Teamwork	218	0.63	3.71	0.51	2.00	5.00	−0.41	0.54
Zest	218	0.78	3.47	0.65	1.80	4.80	−0.32	−0.35

*Top five character strengths of this sample in bold letters (mean > 4.00)*.

**Table 2:** Descriptive statistics of thriving, work engagement, and burnout scales for the whole and tertiary split sample.

**Measure**	***N***	***M***	***SD***	**Min**.	**Max**.
CIT	218	3.85	0.43	2.48	4.76
CIT low	72	3.37	0.29	2.48	3.72
CIT mid	73	3.89	0.09	3.72	4.04
CIT high	73	4.28	0.18	4.04	4.76
WE	218	3.66	1.10	0.22	5.78
WE low	72	2.37	0.64	0.22	3.11
WE mid	73	3.78	0.29	3.11	4.22
WE high	73	4.81	0.40	4.22	5.78
EE	218	2.36	0.95	0.11	4.67
EE low	72	1.31	0.48	0.11	2.00
EE mid	73	2.39	0.24	2.00	2.78
EE high	73	3.39	0.52	2.89	4.67
DP	218	1.48	0.78	0.00	4.20
DP low	72	0.61	0.29	0.00	1.00
DP mid	73	1.41	0.24	1.00	1.80
DP high	73	2.35	0.43	1.80	4.20
rPA	218	3.69	0.50	2.00	5.00
rPA low	72	3.16	0.28	2.00	3.43
rPA mid	73	3.67	0.13	3.43	3.86
rPA high	73	4.24	0.27	3.86	5.00

*CIT, comprehensive inventory of thriving; WE, work engagement; EE, emotional exhaustion; DP, depersonalization; rPA, reduced personal accomplishment*.

### Quantitative Results

Results of the correlation analyses for the whole sample are shown in Table 3. Pearson's correlations of the 24 character strengths with thriving did show 22 significant correlations ranging between *r* = 0.14 (*bravery*) and *r* = 0.67 (*hope*). *Humility* and *spirituality* were not significantly correlated with thriving mean scores. Work engagement highly correlated with *zest* (*r* = 0.67) and had the lowest relations with *kindness* (*r* = 0.13). Concerning the three components of burnout, (a) emotional exhaustion was significantly negatively correlated with five character strengths ranging from *r* = −0.17 (*love*) to *r* = −0.47 (*zest*), (b) depersonalization had the highest negative association with *perseverance* and *leadership* (*r* = −0.27) but was significantly positively correlated with *humor* (*r* = 0.14), and for (c) reduced personal accomplishment, significant negative correlations ranged from *humor* (*r* = −0.14) to *bravery* (*r* = −0.39).

**Table 3:** Pearson's correlation of the 24 character strengths with thriving, work engagement, and burnout questionnaires for the whole sample.

**Character strength**	**CIT**	**WE**	**EE**	**DP**	**rPA**
Appreciation of beauty	0.18^**^	0.07	0.11	0.02	−0.10
Bravery	0.14^*^	0.19^**^	−0.01	−0.014	−0.39^**^
Creativity	0.17^**^	0.21^**^	−0.05	−0.03	−0.21^**^
Curiosity	0.49^**^	0.35^**^	−0.22^**^	−0.03	−0.23^**^
Fairness	0.24^**^	0.10	0.01	−0.24^**^	−0.20^**^
Forgiveness	0.22^**^	0.11	−0.06	−0.05	−0.10
Gratitude	0.38^**^	0.20^**^	−0.06	−0.02	−0.13
Honesty	0.35^**^	0.14^*^	0.03	−0.23^**^	−0.28^**^
Hope	0.67^**^	0.49^**^	−0.32^**^	−0.08	−0.29^**^
Humility	0.03	0.03	0.10	−0.17^*^	−0.08
Humor	0.25^**^	0.25^**^	−0.11	0.14^*^	−0.14^*^
Judgment	0.18^**^	0.14^*^	0.03	−0.03	−0.16^*^
Kindness	0.26^**^	0.13^*^	0.07	−0.02	−0.16^*^
Leadership	0.22^**^	0.15^*^	−0.06	−0.27^**^	−0.32^**^
Love	0.51^**^	0.17^*^	−0.17^*^	−0.05	−0.11
Love of learning	0.29^**^	0.33^**^	−0.19^**^	−0.25^**^	−0.21^**^
Perseverance	0.30^**^	0.21^**^	−0.05	−0.27^**^	−0.29^**^
Perspective	0.23^**^	0.26^**^	−0.08	0.12	−0.19^**^
Prudence	0.21^**^	0.19^**^	−0.02	−0.11	−0.18^**^
Self-regulation	0.17^*^	0.15^*^	−0.01	−0.14^*^	−0.19^**^
Social intelligence	0.27^**^	0.17^*^	0.04	0.01	−0.26^**^
Spirituality	0.002	−0.01	0.11	0.01	0.12
Teamwork	0.34^**^	0.23^**^	−0.08	−0.17^*^	−0.17^*^
Zest	0.64^**^	0.67^**^	−0.47^**^	−0.10	−0.36^**^

*Corrected by Benjamini–Hochberg procedure according to number of predictor variables (n = 24); CIT, comprehensive inventory of thriving; WE, work engagement; EE, emotional exhaustion; DP, depersonalization; rPA, reduced personal accomplishment*;

* *p ≤ 0.05*;

** *p ≤ 0.01(one-tailed)*.

Table 4 shows the results of the correlation analyses between the character strengths and all outcome variables for the tertiary split. Within the (a) low-level group of thriving significant correlations were found with *zest* (*r* = 0.43) and *hope* (*r* = 0.41). In the (b) mid-level group one correlation with thriving remained significant (z*est*; *r* = 0.33). *Spirituality* was the only character strength being significantly negatively correlated with thriving within the (c) high-level group (*r* = −0.28), whereas *love* had the strongest significant positive association (*r* = 0.40) besides *creativity* (*r* = 0.28) and *hope* (*r* = 0.27). Looking at the correlations between the character strengths and work engagement, within the (a) low-level group, they were significantly associated with *zest* (*r* = 0.35), within the (b) mid-level group with *love of learning* (*r* = 0.36), and within the high-level group with *zest* (*r* = 0.37) and *love of learning* (*r* = 0.36). Within the (a) low-level and (b) mid-level group of emotional exhaustion, no significant correlations were found. Only in the (c) high-level group, z*est* (*r* = −0.35) was significantly associated with emotional exhaustion. Concerning the (a) low-level group, values of depersonalization were not significantly in correlation with any of the character strengths. However, moderate correlations were found for *love* (*r* = 0.33) within the (b) mid-level group, and within the (c) high-level group, one significant negative correlation was evident concerning *forgiveness* (*r* = −0.34). Concerning reduced personal accomplishment, only one correlation with *zest* (*r* = −0.31) was significant within the (a) low-level group.

**Table 4:** Pearson correlations of the 24 character strengths with thriving, work engagement, and burnout scales with the tertiary split sample.

**Measure**	**CIT**			**WE**			**EE**			**DP**			**rPA**		
	**Low^a^**	**Mid^b^**	**High^c^**	**Low^a^**	**Mid^b^**	**High^c^**	**Low^a^**	**Mid^b^**	**High^c^**	**Low^a^**	**Mid^b^**	**High^c^**	**Low^a^**	**Mid^b^**	**High^c^**
Appreciation of beauty	−0.01	0.05	0.13	−0.10	−0.07	0.08	0.03	0.01	0.09	0.02	−0.08	−0.01	−0.09	−0.10	0.03
Bravery	−0.03	0.19	0.16	−0.001	0.07	0.24	−0.15	−0.08	0.01	−0.15	−0.17	−0.21	−0.17	−0.19	−0.26
Creativity	0.15	0.10	0.28*	0.11	0.07	0.14	−0.20	0.02	0.16	−0.06	−0.01	0.07	−0.09	−0.15	−0.05
Curiosity	0.24	0.18	0.29	0.01	0.07	0.26	−0.14	−0.25	−0.04	−0.04	0.17	−0.06	−0.24	0.03	−0.02
Fairness	0.14	0.06	0.07	−0.14	0.07	−0.02	−0.09	0.12	0.17	0.04	−0.04	−0.20	−0.28	−0.32	−0.17
Forgiveness	0.05	−0.07	−0.10	−0.18	0.17	0.07	0.05	0.07	0.09	0.22	0.26	−0.34**	−0.21	−0.21	0.02
Gratitude	0.24	0.08	−0.003	−0.09	0.07	−0.03	−0.03	−0.03	−0.06	−0.02	0.09	−0.06	−0.18	−0.08	0.20
Honesty	0.20	0.01	0.02	−0.17	0.10	0.03	−0.09	0.04	0.22	0.01	−0.07	−0.04	−0.16	−0.15	−0.29
Hope	0.41**	0.23	0.27*	0.26	0.10	0.001	−0.05	−0.12	−0.15	−0.05	0.25	−0.05	−0.24	−0.05	0.05
Humility	−0.05	−0.05	0.02	−0.15	−0.05	−0.06	0.10	0.10	0.15	−0.04	−0.20	−0.11	−0.07	−0.18	−0.11
Humor	−0.04	0.15	0.16	−0.05	−0.13	0.08	−0.26	0.08	−0.06	0.14	0.003	0.16	−0.02	0.03	−0.26
Judgment	0.07	−0.02	0.11	−0.18	−0.01	−0.12	−0.09	−0.01	0.19	−0.21	−0.02	0.003	0.18	0.08	−0.05
Kindness	0.13	−0.14	0.16	−0.02	0.02	0.11	−0.02	0.18	0.19	0.14	−0.07	0.01	−0.16	−0.27	−0.16
Leadership	0.09	−0.04	0.09	−0.18	0.04	0.11	−0.14	−0.18	0.12	−0.12	−0.02	−0.17	−0.05	−0.28	−0.23
Love	0.25	0.18	0.40**	−0.13	0.11	0.06	−0.08	−0.28	0.15	−0.004	0.33**	0.14	−0.11	−0.15	0.12
Love of learning	0.10	0.19	0.30	−0.11	0.36**	0.36**	−0.19	0.03	0.02	−0.19	0.02	−0.06	−0.24	−0.11	0.13
Perseverance	0.26	−0.06	−0.06	−0.04	0.06	0.09	−0.02	−0.07	0.14	−0.16	0.01	−0.22	−0.25	−0.06	−0.18
Perspective	0.09	−0.04	−0.07	0.05	0.17	−0.01	−0.05	−0.14	−0.06	−0.10	0.10	0.15	0.03	0.22	0.13
Prudence	0.12	−0.13	0.09	−0.11	0.08	0.08	−0.11	−0.02	0.09	−0.08	−0.01	−0.06	0.01	−0.04	−0.12
Self-Regulation	0.19	−0.06	0.10	−0.13	0.13	0.02	−0.09	−0.18	0.11	−0.12	0.07	−0.07	−0.14	−0.05	0.01
Social Intelligence	0.12	−0.13	0.06	0.03	0.06	0.04	−0.11	−0.02	0.10	0.10	−0.05	−0.03	−0.08	−0.08	−0.14
Spirituality (–)	0.004	0.15	−0.28*	−0.15	0.08	−0.09	0.15	0.13	0.09	0.13	0.03	0.10	0.02	−0.16	0.22
Teamwork	0.26	−0.06	−0.02	−0.18	0.19	0.03	0.03	−0.11	0.07	0.14	0.02	−0.10	−0.26	−0.30	−0.05
Zest	0.43**	0.33**	0.21	0.35**	0.29	0.37**	−0.13	−0.22	−0.35**	0.003	0.18	−0.13	−0.31**	−0.03	0.01

*Corrected by Benjamini–Hochberg procedure according to the number of predictor variables (n = 24); CIT, comprehensive inventory of thriving; WE, work engagement; EE, emotional exhaustion; DP, depersonalization; rPA, reduced personal accomplishment*.

a *n = 72*.

b *n = 73*.

c *n = 73*.

Bold* = p ≤ 0.05.

Bold** = p ≤ 0.01 (one-tailed).

### Qualitative Results

Table 5 depicts the answers to the question by resident physicians and medical specialist educators. As shown in the respective table, resident physicians mentioned *social intelligence* and *teamwork* (each 3x) followed by *fairness* (2x), *perseverance* (2x), and *honesty* (2x) to be the most important for their well-being at work. All medical specialist educators mentioned the character strength, *humility* (4x) followed by *teamwork* (3x), *kindness* (2x), *social intelligence* (2x), and *zest* (2x) to be the most relevant for the well-being of resident physicians at work.

**Table 5:** Answers to the interview question: *In your opinion, which out of these 24 character strengths are the five most important a resident physician in the hospital needs to feel well*?

**Character strengths**	***N total***	**RP**	**MSE**
**Teamwork**	**6**	3	3
**Humility**	**5**	1	4
**Social intelligence**	**5**	3	2
Fairness	3	2	1
Honesty	3	2	1
Kindness	3	1	2
Perseverance	3	2	1
Judgment	2	1	1
Curiosity	2	1	1
Zest	2	–	2
Hope	1	–	1
Humor	1	1	–
Love of learning	1	–	1
Self-regulation	1	1	–

*RP, resident physicians; MSE, medical specialist educators. Bold values three most frequently mentioned character strenghts in total*.

### Combining Quantitative and Qualitative Results

Bringing questionnaire results and interview-gathered opinions together, differences appeared in terms of character strengths being apparently relevant for the well-being of resident physicians at work. Although *humility* was most frequently mentioned by medical specialist educators, resident physicians did not consider this character strength as important as others. Only one significant low negative correlation (*r* = −0.17) between *humility* and the total mean of depersonalization was found. Regarding all tertiary split outcome groups, no significant correlation with *humility* was evident within any group. *Teamwork*, according to the personally asked question, was equally important for resident physicians and medical specialist educators. Results of the quantitative analyses revealed low to moderate correlations for *teamwork* with thriving (*r* = 0.34), work engagement (*r* = 0.23), depersonalization, and reduced personal accomplishment (both *r* = −0.17). Tertiary outcome splits revealed no significant correlations for *teamwork* with any outcome variable. *Social intelligence*, which is rated as the highly important character strength by resident physicians and medical specialist educators, did show only low correlations with thriving (*r* = 0.27), work engagement (*r* = 0.17), and reduced personal accomplishment (*r* = −0.26). Regarding the tertiary splits, no significant correlations were found for *social intelligence* at all.

## Discussion

This study was carried out in order to illuminate whether there are specific character strengths among medical professionals that are particularly relevant to work-related well-being. Thereby, specific attention was paid to the entire spectrum of well-being. The combination of quantitative and qualitative data enabled an identification of different aspects regarding the relationship of character strengths and work-related well-being. In particular, the character strengths, such as *hope* and *zest* correlated highly with the well-being outcome variables. However, when adding qualitative data, resident physicians and medical specialist senior educators assigned higher importance to *humility, social intelligence*, and *teamwork*. Correlations between character strengths and thriving, work engagement, and the burnout dimensions were not significant across the entire continuum of the respective measurement scales, indicating distinct effects of some character strengths at different levels of well-being.

### Relevance of Character Strengths for Well-Being

The first two hypotheses focused on whether there are specific character strengths correlating significantly higher with the well-being of resident physicians at work than others and whether this is different across the entire continuum of thriving, work engagement, and burnout. Throughout, the highest correlations were not only found for the character strengths, such as *hope* and *zest* with thriving, confirming the studies of Hausler et al. (2017b), but also with work engagement and emotional exhaustion. Both character strengths belong to the so-called “happiness strengths,” being in particular associated with life and occupational satisfaction, engagement, and work meaningfulness in some samples (Harzer and Ruch, 2013; Littman-Ovadia et al., 2016). *Hope* and *zest* had the highest negative associations with emotional exhaustion, while *leadership* and *perseverance* had the highest negative correlation with depersonalization, and *bravery* and *zest* with reduced personal accomplishment. Drawing the attention to the type of the character strengths associated with the different scales, it is noticeable that those having the highest correlations with thriving, work engagement, and emotional exhaustion can be assigned to “tonic strengths” (e.g., *hope, curiosity*, and *zest*) whereas some “phasic strengths” (e.g., *bravery* and *leadership*) were more strongly associated with depersonalization and reduced personal accomplishment. The less frequent expression of phasic strengths might play a role here. The context in which physicians find themselves could determine whether the corresponding character strength can be applied at work or not. Hence, it can have a weaker or stronger influence on individual components affecting the well-being compared to tonic strengths (Huber et al., 2020).

When splitting the outcome variables into thirds, *zest* was the only character strength correlating with outcomes across all the outcome-level groups (low- and mid-level thriving, low- and high-level work engagement, high-level emotional exhaustion, and low-level reduced personal accomplishment). This suggests that *zest* is more related to the entire spectrum of work-related well-being than others. It could be a core character strength candidate for future interventions. In contrast, *forgiveness* was solely significantly related in the high-level group of depersonalization, indicating a specific relevance. Depersonalization of hospital physicians is mainly characterized by a rather careless or unemotional treatment of patients. This might be due to personal and environmental demands at work and managers being responsible for the system. Therefore, particularly *forgiveness* could make a positive contribution to higher-level depersonalization as it mainly means “to let go.” In many cases, this is the letting go of frustration, disappointment, resentment, or other painful feelings associated with an offense and involves accepting the shortcomings, flaws, and imperfections of others (or circumstances) and giving them another chance. One study showed that distress about “administrative burden and academic stress” as well as “distress about coworkers” remained significant independent predictors of higher burnout scores (Weintraub et al., 2020) also arguing for *forgiveness* to letting bygones be bygones. “Inverted patterns” were also found for *creativity* and *spirituality* in terms of significant correlations exclusively with the high-level group of thriving, whereas *love* had a significant correlation to mid-level depersonalization. *Creativity* was positively associated with high levels of thriving, thus indicating that originality and adaptiveness, thinking of new ways to do things, producing original ideas or behaviors is depending on an individuals' feeling as well. Possibly, when thriving is not that distinct, hospital physicians cannot access their *creativity* adequately as they have got their hands full complying with the “basic” medical challenges and stressors while only less resources are left to conceptualize something useful new. Interestingly, *spirituality* was negatively associated with the high-level group of thriving. In another study, disentangling thriving, in particular the subscale “autonomy” was decisive for this negative relation (Huber et al., 2020). Autonomy (control) is defined by life decisions on one's own responsibility, belief in one's personal skills, and the internal locus of control being somehow contrary to the definition of *spirituality*. This character strength comprises aspects like life calling, beliefs about the universe, and practices that connect with the transcendent (“sacred”) which is blessed, holy, or particularly special (secular or non-secular). It involves the belief that there is a dimension to life beyond human understanding. Today's medical culture teaches physicians to quickly develop self-confidence and to move beyond all insecurities. Furthermore, qualities as technical skills and medical knowledge are often higher evaluated than virtues or other “non-technical” skills. Therefore, one could assume that the understanding of thriving in today's hospital physicians collides with *spirituality*, in particular when having higher values. Interestingly, this significant negative correlation would not have been detected without the tertiary split, as there is no significant correlation with thriving in total.

The positive association of *love* to the mid-level group of depersonalization seems particularly fascinating. Depersonalization of hospital physicians can also be interpreted to some degree as defense mechanism or strategy to cope with all the stressors and issues of patients to remain capable for work. Physicians may be generally at a greater risk for this burnout symptom as scientific reasoning teaches one to look for what is wrong with a situation or person that is, to search for “disease” rather than appreciating what is working well (Restauri et al., 2019). *Love* as a character strength refers to the degree to which one values close relationships with people, and contributes to that closeness in a warm and genuine way. Low compassionate *love* may lead to missing patient commitment resulting in the fact that no depersonalization is needed to “protect” oneself while high *love* may lead to the feeling that depersonalization is not allowed as oneself is totally involved. However, when hospitals' physicians are located in the middle, *love* might increase the need of depersonalization to manage everyday life in the hospital for longer periods. Finally, *love of learning* was significantly related with the mid-level and high-level group of work engagement. These correlations could indicate that this character strength tends to be relevant in terms of rather higher work engagement, as it comprises a passion for learning, a desire to learn just for learning, a motivation to expand the fund of knowledge and a desire to hold on to and deepen that information. Thus, *love of learning* complements hospital physicians' higher work engagement quite well as their strong involvement in and concentration on work tasks is often associated with having a huge medical knowledge.

One very cautious conclusion might be that some character strengths tend to unfold their importance rather at the foundation of the well-being of the resident physicians, whereas others do that at or toward a higher level of “flow” or overall satisfaction. Again, the intention here is not to “diagnose” or differentiate between “low or high burnout” in a clinical sense but to provide a deeper understanding of different patterns character strengths might have for the graduations of work-related well-being in the context of medical staff. A recent study demonstrated significant positive effects of thriving on the applicability of signature character strengths at later time points indicating that higher levels of well-being might be mandatory first to have access to one's own signature character strengths in general, supporting this preliminary observation (Huber et al., 2021). Another study by Strecker et al. (2019) pursuing this approach, indicated that the higher the applicability of signature character strengths at hospital physician's work, the higher may be the positive reaction of social support at work by colleagues and supervisors. Drawing these studies together, this positive reaction could be explained by the unfolding certain applied character strengths at different levels of well-being that are relevant in the present work setting and encouraged by its surroundings. Taking a comprehensive look at the tertiary split outcomes, significant correlations considering all constructs and each of their respective meaning, the aforementioned special potential effects when reporting lower well-being at work may support the importance in particular of *forgiveness* in a hospital setting as fundamental character strengths (in analogy with Maslow's pyramid of needs), whereas *creativity* and *love of learning* seem to unfold their importance rather at higher levels of well-being at work. *Zest*, being apparently more independent from the subjective level reported than others, could represent the core character strength for future overall interventions.

People acting not accordingly or against their own preferences are naturally less likely to be happy at the workplace as a basic person-condition-fit is not ensured. In general, interpersonal skills are underrepresented in medical curricula, whereas the training of technical skills is heavily emphasized (Montgomery et al., 2013). Furthermore, the professional identity of future physicians is shaped during medical school (Hafferty, 1998; Elliott et al., 2009). Students experience a learning environment also characterized by the hidden curriculum, which comprises aspects, such as rituals or customs that are taken for granted (Hafferty, 1998). This hidden curriculum is a huge part of the learning experience and values, performance-oriented or competitive behavior, therefore opposed to character strengths like *forgiveness*. The current medical school system (beginning with an admission test) rather supports individualistic, cognitive- and performance-oriented working (Thiel, 2015) being simultaneously very demanding and causing above-average depressive and burnout symptoms, health concerns, and impaired well-being, also during residency (Dyrbye et al., 2014; Dyrbye and Shanafelt, 2016).

### Adding Qualitative Data From the Content Analysis

Taking all interview answers together, differences were found in the importance of ranking of character strengths regarding the well-being of the resident physicians. Out of the 14 mentioned character strengths in total, 9 were chosen at least once in both groups (*curiosity, fairness, honesty, humility, judgment, kindness, perseverance, social intelligence*, and *teamwork*). Concerning the most frequently mentioned character strengths, the highest (moderate) correlation was found for *teamwork* and thriving, whereas only low correlations revealed for *humility* and *social intelligence*. Higher correlations were found for the less mentioned character strengths (e.g., *hope, curiosity, love of learning*, or *zest*). Tertiary split results did not show any significant correlation for *humility* with respective outcome variables, whereas *humility* was valued important by all medical specialist senior educators (vs. once by a resident physician). *Humility* is defined by accurately evaluating your accomplishments, being aware of your mistakes and gaps in knowledge, not seeking the spotlight or other's attention, and not regarding yourself as being special (Seligman, 2011). In most cases, medical specialist educators possess much more work experience than resident physicians do. Therefore, one could assume that the opinion of medical specialist educators could have been based on everyday hospital life experiences they have made throughout their career and their own residency. They have possibly understood *humility* rather to be a characteristic resident physicians “should” have in order to adapt to the hierarchical system in hospitals (maybe also in terms of, e.g., not demanding great deals, not expecting too much, being content with the command structure) instead of a characteristic they “need” to have to feel well. The different roles and hierarchical levels in a hospital that resident physicians and medical specialist senior educators hold might play a role as well. The hierarchical system in hospitals originated from formations during the last century. Hierarchical processes and principles of military treatment (“triage”) have proved their worth during World War I, so they were maintained during World War II and lingered afterwards. Hospitals and even medical education itself still adapt continuously to changing requirements, societal needs, and expectations (Maniate, 2017), but former hierarchical (military) structures remained more or less in clinical daily routine. In addition to its justified functionality, this hierarchical system has its drawbacks. It is characterized by less contact and less communication on equal terms in everyday working life, which might subsequently lead to different perceptions due to different viewpoints and hierarchical levels. Maybe resident physicians would not have thought that medical specialist senior educators named, for example, *zest* as being important for their well-being, because they never communicate about those aspects in the stressful hierarchically organized everyday duty. Another perspective can be adopted by taking a closer look at the two interviewed generations as differences among them might be due to modified motivational aspects and values shaping their work and life environment (Duffrin et al., 2016). The four interviewed resident physicians were on average 33 years old, therefore belonging to the “new” *Generation Y*, being mostly classified as self-centered, naturally demanding greater deals, overly self-confident, seeking for permanent feedback and appreciation but suffering there from (Myers and Sadaghiani, 2010). In contrast to the medical specialist senior educators being 51 years on average (*Generation X:* convincing through competence, highly loyal, yielding power, and responsibility to others; Mörstedt, 2016), the younger *Generation Y* does not perceive *humility* as being important for their well-being at work as this character strength would not match their beliefs. Linley et al. (2007) found significant positive correlations between age and strengths of temperance, which *humility* is belonging to. Of course, belonging to either one or the other generation does not automatically mean that the respective physicians share the same characteristics, but it could be one possible explanation.

Beyond that generational discourse in terms of differences, some fundamental factors inherent to the medical field remained to be important over generations (Duffrin et al., 2016). For example, *teamwork* (being mentioned three times by resident physicians and medical specialist senior educators each) means that in team situations, you contribute to the team's success being a dedicated and loyal member, valuing the group goals and purposes, and respecting those who are rightfully in positions of authority (Seligman, 2011). In terms of being a physician, the team's goal or success since decades has been saving the patient by all means. Although working in more or less hierarchical interdisciplinary teams including various professions can also entail challenges and strains (e.g., many agreements, potential inter-individual conflicts, disengagement, and unclear goals,) it has mostly been supportive to achieve the common purpose by discussing medical decisions within the team, gathering all relevant information, following instructions of trustful authorities, or obeying standardized procedures. In other words, the working conditions in a hospital simply do require working together as a team; otherwise work cannot be done effectively (O'Leary et al., 2012). As a result, cooperation in teams is constantly required and encouraged, which means that this strength can constantly be developed. From this perspective, *teamwork* can be interpreted as phasic strength with frequent application in the context of the hospital. Thus, both young resident physicians and medical specialist educators considered *teamwork* to be highly important for the well-being at work, representing the most frequently mentioned character strength in total. *Teamwork* also showed the highest accordance with quantitative data, although the correlations did not exceed moderate size.

The most frequently mentioned character strength by resident physicians besides *teamwork* was *social intelligence* (ranked third by medical specialist educators). In terms of the VIA-definition, *socially intelligent* persons are more likely to know how other people tick, are able to notice differences among others in particular with respect to their moods, temperament, motivations, and intentions and then act upon these distinctions (Seligman, 2002). This character strength shows up in socially skilled action and can also be interpreted as “emotional intelligence” (Goleman, 1995), being important for respect, communication, or empathy in general (Hertel-Waszak et al., 2017). However, *social intelligence* showed only low significant correlations with thriving, work engagement, and reduced personal accomplishment in total. According to tertiary split results, no significant correlations for *social intelligence* were found at all. One possible explanation for these results might be that within the context of a highly technologized hospital, there are not enough possibilities to apply *social intelligence* to an extent that could directly lead to a perceived work-related sense of achievement (e.g., patients' higher satisfaction or better adherence). To put it bluntly, daily hospital work life as it is today, works without promoting *social intelligence* as patients will most likely survive with or without these respective skills. Like arguing before with regard to *humility*, the interviewed resident physicians might have wanted to state that this character strength should be more important as it currently is.

### Limitations and Future Research

There are several important limitations to this study. It is conceivable that “to feel well” was not understood by all interviewees in the same manner. For example, some might have interpreted the question as what kind of character strengths are important in terms of a general sense of well-being at work, while others might have assumed that the question aimed at what is important for the profession as physician itself. Although the focus was repeatedly emphasized by the interviewer, the answers should be interpreted carefully. Also, the individual understanding of the mentioned character strengths in the interviews and how their correspondents were operationalized by the respective VIA-scale might be different and therefore, not fully comparable. Moreover, since we did not ask resident physicians and medical specialist educators from each of the 16 medical specialties participating in the online survey, a comprehensive evaluation of the perceived importance of character strengths for work-related well-being was not possible. This could have resulted in skewed results concerning character strengths and their relations to thriving, work engagement, and burnout. Maybe, certain character strengths are considered important in one medical specialty, and not in others. Nevertheless, the medical departments for the interviews have been chosen with regard to the best possible representation of medical disciplines in general and the frequency of the medical disciplines of the respondents. Concerning the VIA-IS questionnaire, reliability might be reduced due to partially low Cronbach's alpha values of certain VIA-scales (e.g., *teamwork* and *perspective*). This can result in the corresponding constructs not being measured distinctively, thus being distorted. Furthermore, low reliability may lead to a loss in power, which in turn can result in an increased probability of type II error (Hopkins and Hopkins, 1979). Using the one-dimensional short version measuring work engagement leads to a lack of comparability with the three burnout scales; therefore the long version of the instrument might be better suited for future studies.

The tertiary split of the outcome variables to detect more detailed differences, resulted in small standard deviations. In particular, within the mid-level groups, smaller variances were evident compared to the other two groups. Of course, splitting outcome variables into sub-groups naturally implies producing smaller variances within the respective groups compared to the whole sample, but this circumstance is important when interpreting the tertiary results. Smaller variances increase the likelihood for smaller (thus non-significant) correlations, consequently leading to an underestimation of true relations. The “variance-restriction” means that the “effect” of a certain character strength on an outcome (e.g., thriving) depends on the respective classification (low-, mid-, high-level group) because the variance of the predictor or dependent variable changes as a function of the moderator. An alternative approach to extract “the” most important character strength for the well-being of the resident physicians at work, however, was not feasible as there is no “pure” character strength and various combinations and characteristics of them influence and belong to each other. Therefore, it would have been necessary to control for all the other 23 character strengths also eliminating variance. It is conceivable that due to the limited response rate in the quantitative sample and the small numbers of qualitative interviews, specific unobserved characteristics of a selective sample were recorded that are not relevant in a larger study group. It is also important to note that the cross-sectional study design cannot provide explanations about causal relationships (cause/effect). In particular, due to the trait and state debate, respectively phasic and tonic properties of the character strengths, it is reasonable that they could change over time, which can lead to a shift in the relationships described here.

From the results of this study, different theoretical and practical implications can be derived for future projects concerning character strengths and well-being outcomes in the context of hospital physicians. On a theoretical level, upcoming research should focus on the entire spectrum of various well-being outcomes. It should be examined whether the character strengths, which have turned out to be relevant, are important over an expanded timespan. Replication of these findings in other hospitals or working environments with a cross-cultural perspective (e.g., Europe and Asia) can be important in order to ensure that the results are generalizable. On a practical level, future experimental studies could test on interventions targeting *forgiveness* (when individuals report high depersonalization) or *creativity* and *love of learning* (when individuals report high levels of thriving or work engagement) to replicate the novel findings of this study. In general, future studies may consider how individuals at different levels of well-being might benefit from tailored approaches, not assuming that one intervention fits all levels of well-being. Therefore, a positive psychology-based coaching intervention, including the “Workplace PERMA Profiler” (Butler and Kern, 2016), “Gratitude Reflections,” “Best Self,” and “Using Strengths in New Ways” interventions might be suitable, which have shown to reduce burnout and increase work engagement effectively (McGonagle et al., 2020). In particular, *gratitude* can flip the “disease search pattern” of physicians causing depersonalization by fostering meaningfulness (Restauri et al., 2019). The ability to cultivate a sense of meaning in work, also postulated in the PERMA-model, is a critical connection between engagement on the one hand and burnout on the other as meaning might be a defining factor choosing to continue to pursue career goals (Larsen et al., 2021). Tailored interventions could also focus on the reflection of virtues in “good doctors” (Arthur et al., 2015) or further implement mindfulness-based strengths practice into medical curricula and training of physicians (Niemiec, 2014).

## Conclusion

Quantitative data of this study revealed that the character strengths of *hope* and *zest* were particularly highly correlated with the well-being outcome variables. Resident physicians and medical specialist educators however assigned high importance to *humility, social intelligence*, and *teamwork*. These differences may be driven by different work experiences, professional understandings, generational beliefs, or social expectations. Correlations between character strengths and thriving, work engagement, emotional exhaustion, depersonalization, or reduced personal accomplishment were not significant across the entire continuum of the respective measurement scales, indicating distinct effects of some character strengths at different levels of well-being. Therefore, interventions tailored for respective outcome levels warrant further research.

## Data Availability Statement

The raw data supporting the conclusions of this article will be made available by the authors, without undue reservation.

## Ethics Statement

This study was conducted in accordance with recommendations of The Board for Ethical Questions in Science of the University of Innsbruck including written informed consent from all subjects. All participants gave written informed consent subject to the regulations of the Declaration of Helsinki. The protocol was permitted by The Board for Ethical Questions in Science of the University of Innsbruck.

## Author Contributions

AH, CS, TH, and SH were substantially involved in planning and conducting the study. AH and TK are equally contributing first authors, they drafted the manuscript and carried out the data analysis. All authors revised the manuscript critically for important intellectual content, read, and approved the submitted version.

## Conflict of Interest

The authors declare that the research was conducted in the absence of any commercial or financial relationships that could be construed as a potential conflict of interest.

## References

[B1] Allport G. W. (1937). Personality: A Psychological Interpretation. Oxford: Holt.

[B2] Angerer P. Weigl M. (2015). Physicians' psychosocial work conditions and quality of care: a literature review. Profess. Professional. 5, 1–20. 10.7577/pp.960

[B3] Arnetz B. B. (2001). Psychosocial challenges facing physicians of today. Soc. Sci. Med. 52, 203–213. 10.1016/S0277-9536(00)00220-3 11144776

[B4] Arthur J. Kristjánsson K. Thomas H. Kotzee B. Ignatowicz A. Qui T. (2015). Virtuous Medical Practice: Research Report. University of Birmingham, Birmingham.

[B5] Bakker A. Demerouti E. (2007). The job demands-resources model: state of the art. J. Manage. Psychol. 22, 309–328. 10.1108/02683940710733115

[B6] Bazzoli G. J. Clement J. P. Lindrooth R. C. Chen H.-F. Aydede S. K. Braun B. I.. (2007). Hospital financial condition and operational decisions related to the quality of hospital care. Med. Care Res. Rev. 64, 148–168. 10.1177/1077558706298289 17406018

[B7] Benjamini Y. Hochberg Y. (1995). Controlling the false discovery rate: a practical and powerful approach to multiple testing. J. R. Stat. Soc. Ser. B 57, 289–300.

[B8] Bohman B. D. Dyrbye L. Sinsky C. A. Linzer M. Olson K. Babbott S.. (2017). Physician Well-Being: The Reciprocity of Practice Efficiency, Culture of Wellness, and Personal Resilience. Available online at: https://catalyst.nejm.org/physician-well-being-efficiency-wellness-resilience (accessed April 23, 2021).

[B9] Büssing A. Perrar K.-M. (1992). Die Messung von Burnout: Untersuchung einer deutschen Fassung des Maslach Burnout Inventory (MBI-D) [The measurement of burnout: investigating the German version of the Maslach Burnout Inventory (MBI-D)]. Diagnostica 38, 328–353. 8004334

[B10] Butler J. Kern M. L. (2016). The PERMA-Profiler: a brief multidimensional measure of flourishing. Int. J. Wellbeing 6, 1–48. 10.5502/ijw.v6i3.526

[B11] Cockerham W. C. (2014). Medical Sociology. John Wiley and Sons, Ltd. Retrieved from: https://www.healthknowledge.org.uk/public-health-textbook/medical-sociology-policy-economics/4b-health-care/section9

[B12] Duffrin C. Cashion M. Cummings D. M. Whetstone L. Firnhaber J. Levine G.. (2016). Generational differences in practice site selection criteria amongst primary care physicians. Marshall J. Med. 2:9. 10.18590/mjm.2016.vol2.iss1.9

[B13] Dunn L. B. Iglewicz A. Moutier C. (2008). A conceptual model of medical student well-being: promoting resilience and preventing burnout. Acad. Psychiatry 32, 44–53. 10.1176/appi.ap.32.1.44 18270280

[B14] Dyrbye L. Shanafelt T. (2016). A narrative review on burnout experienced by medical students and residents. Med. Educ. 50, 132–149. 10.1111/medu.12927 26695473

[B15] Dyrbye L. N. West C. P. Satele D. Boone S. L. Tan L. Sloan J.. (2014). Burnout among U.S. medical students, residents, and early career physicians relative to the general U.S. population. Acad. Med. 89, 443–451. 10.1097/ACM.0000000000000134 24448053

[B16] Elliott D. D. May W. Schaff P. B. Nyquist J. G. Trial J. Reilly J. M.. (2009). Shaping professionalism in pre-clinical medical students: professionalism and the practice of medicine. Med. Teach. 31, e295–e302. 10.1080/01421590902803088 19811137

[B17] Freidlin P. Littman-Ovadia H. andNiemiec R. M. (2017). Positive psychopathology: social anxiety via character strengths underuse and overuse. Pers. Individ. Differ. 108, 50–54. 10.1016/j.paid.2016.12.003

[B18] Gelman A. Park D. K. (2009). Splitting a predictor at the upper quarter or third and the lower quarter or third. Am. Stat. 63, 1–8. 10.1198/tast.2009.0001

[B19] Goldzweig C. L. Towfigh A. Maglione M. Shekelle P. G. (2009). Costs and benefits of health information technology: new trends from the literature. Health Affairs 28, w282–w293. 10.1377/hlthaff.28.2.w282 19174390

[B20] Goleman D. (1995). Emotional Intelligence. New York, NY: Bantam.

[B21] Hafferty F. W. (1998). Beyond curriculum reform: confronting medicine's hidden curriculum. Acad. Med. 73, 403–407. 10.1097/00001888-199804000-00013 9580717

[B22] Hahn S. Zeller A. Needham I. Kok G. Dassen T. Halfens R. J. G. (2008). Patient and visitor violence in general hospitals: a systematic review of the literature. Aggress. Viol. Behav. 13, 431–441. 10.1016/j.avb.2008.07.001

[B23] Harzer C. Ruch W. (2013). The application of signature character strengths and positive experiences at work. J. Happ. Stud. 14, 965–983. 10.1007/s10902-012-9364-0

[B24] Hausler M. Huber A. Strecker C. Brenner M. Höge T. Höfer S. (2017a). Validierung eines Fragebogens zur umfassenden Operationalisierung von Wohlbefinden. Die deutsche Version des Comprehensive Inventory of Thriving (CIT) und die Kurzversion Brief Inventory of Thriving (BIT) [Validation of a holistic measure for the construct of well-being: the German version of the Comprehensive Inventory of Thriving (CIT) and the short version Brief Inventory of Thriving (BIT)]. Diagnostica 63, 219–228. 10.1026/0012-1924/a000174

[B25] Hausler M. Strecker C. Huber A. Brenner M. Höge T. Höfer S. (2017b). Associations between the application of signature character strengths, health and well-being of health professionals. Front. Psychol. 8:1307. 10.3389/fpsyg.2017.01307 28824492 PMC5534439

[B26] Hausler M. Strecker C. Huber A. Brenner M. Höge T. Höfer S. (2017c). Distinguishing relational aspects of character strengths with subjective and psychological well-being. Front. Psychol. 8:1159. 10.3389/fpsyg.2017.01159 28744245 PMC5504157

[B27] Hertel-Waszak A. Brouwer B. Schönefeld E. Ahrens H. Hertel G. Marschall B. (2017). Medical doctors' job specification analysis: a qualitative inquiry. GMS J. Med. Educ. 34:43. 10.3205/zma001120 29085887 PMC5654118

[B28] Höfer S. Hausler M. Huber A. Strecker C. Renn D. Höge T. (2019). Psychometric characteristics of the german values in action inventory of strengths 120-item short form. Appl. Res. Qual. Life 15, 597–611. 10.1007/s11482-018-9696-y 32457816 PMC7250639

[B29] Höge T. Strecker C. Hausler M. Huber A. Höfer S. (2019). Perceived socio-moral climate and the applicability of signature character strengths at work: a study among hospital physicians. Appl. Res. Qual. Life 15, 463–484. 10.1007/s11482-018-9697-x 33304415 PMC7116458

[B30] Hopkins K. D. Hopkins B. R. (1979). The effect of the reliability of the dependent variable on power. J. Spec. Educ. 13, 463–466. 10.1177/002246697901300413

[B31] Huber A. Bair A. Strecker C. Höge T. Höfer S. (2021). Do more of what makes you happy? The applicability of signature character strengths and future physicians' well-being and health over time. Front. Psychol. 10.3389/fpsyg.2021.534983 PMC8200469 34135792

[B32] Huber A. Strecker C. Hausler M. Kachel T. Höge T. Höfer S. (2019). Possession and applicability of signature character strengths: what is essential for well-being, work engagement, and burnout? Appl. Res. Qual. Life 15, 415–436. 10.1007/s11482-018-9699-8 32457814 PMC7250640

[B33] Huber A. Strecker C. Kachel T. Höge T. Höfer S. (2020). Character strengths profiles in medical professionals and their impact on well-being. Front. Psychol. 11:566728. 10.3389/fpsyg.2020.566728 33424679 PMC7786021

[B34] Huber A. Webb D. Höfer S. (2017). The German version of the strengths use scale: the relation of using individual strengths and well-being. Front. Psychol. 8:637. 10.3389/fpsyg.2017.00637 28496424 PMC5406780

[B35] IBM Corp. (2019). IBM SPSS Statistics for Windows, Version 26.0. Armonk, NY: IBM Corp.

[B36] Ishak W. W. Lederer S. Mandili C. Nikravesh R. Seligman L. Vasa M.. (2009). Burnout during residency training: a literature review. J. Grad. Med. Educ. 1, 236–242. 10.4300/JGME-D-09-00054.1 21975985 PMC2931238

[B37] Kachel T. Huber A. Strecker C. Höge T. Höfer S. (2020). Development of cynicism in medical students: exploring the role of signature character strengths and well-being. Front. Psychol. 11:328. 10.3389/fpsyg.2020.00328 32174874 PMC7056910

[B38] Kachel T. Strecker C. Haselgruber T. Höge T. Höfer S. (2019). “Ist ein Soziomoralisches Klima im Krankenhaus möglich? Eine Mixed-Methods Studie zu dessen Ausprägung, Einflussfaktoren und Auswirkungen [Is a sociomoral climate in hospitals possible? A mixed-methods study on its characteristics, influencing factors and effects],” in Hallesche Schriften zur Betriebswirtschaft. Symposium Qualitative Forschung 2018, Vol. 34, eds M. Raich and J. Müller-Seeger (Springer Fachmedien Wiesbaden), 177–197. 10.1007/978-3-658-28693-4_8

[B39] Keyes C. L. M. (2002). The mental health continuum: from languishing to flourishing in life. J. Health Soc. Behav. 43, 207–222. 10.2307/3090197 12096700

[B40] Klein J. Grosse Frie K. Blum K. von dem Knesebeck O. (2010). Burnout and perceived quality of care among German clinicians in surgery. Int. J. Qual. Health Care 22, 525–530. 10.1093/intqhc/mzq056 20935011

[B41] Larsen D. Chu J. T. Yu L. Chang Y. Donelan K. Palamara K. (2021). Correlating burnout and well-being in a multisite study of internal medicine residents and faculty. J. Gen. Intern. Med. 36, 1422–1426. 10.1007/s11606-021-06653-4 33674923 PMC8131435

[B42] Lee R. T. Seo B. Hladkyj S. Lovell B. L. Schwartzmann L. (2013). Correlates of physician burnout across regions and specialties: a meta-analysis. Hum. Resour. Health 11:48. 10.1186/1478-4491-11-48 24074053 PMC3849515

[B43] Linley A. P. Maltby J. Wood A. M. Joseph S. Harrington S. Peterson C.. (2007). Character strengths in the United Kingdom: the VIA inventory of strengths. Pers. Individ. Differ. 43, 341–351. 10.1016/j.paid.2006.12.004

[B44] Littman-Ovadia H. Freidlin P. (2019). Positive psychopathology and positive functioning: OCD, flourishing and satisfaction with life through the lens of character strength underuse, overuse and optimal use. Appl. Res. Qual. Life 15, 529–549. 10.1007/s11482-018-9701-5

[B45] Littman-Ovadia H. Lavy S. Boiman-Meshita M. (2016). When theory and research collide: examining correlates of signature strengths use at work. J. Happ. Stud. 18, 527–548. 10.1007/s10902-016-9739-8

[B46] Maniate J. (2017). Trends and opportunities in medical education: aligning to societal needs and expectations. Arch. Med. Health Sci. 5:154. 10.4103/amhs.amhs_98_17

[B47] Maslach C. Schaufeli W. B. Leiter M. P. (2001). Job burnout. Annu. Rev. Psychol. 52, 397–422. 10.1146/annurev.psych.52.1.397 11148311

[B48] McGonagle A. K. Schwab L. Yahanda N. Duskey H. Gertz N. Prior L.. (2020). Coaching for primary care physician well-being: a randomized trial and follow-up analysis. J. Occup. Health Psychol. 25, 297–314. 10.1037/ocp0000180 32297776

[B49] Mechanic D. (2003). Physician discontent: challenges and opportunities. JAMA 290, 941–946. 10.1001/jama.290.7.941 12928472

[B50] Montgomery A. Todorova I. Baban A. Panagopoulou E. (2013). Improving quality and safety in the hospital: the link between organizational culture, burnout, and quality of care. Br. J. Health Psychol. 18, 656–662. 10.1111/bjhp.12045 23607495

[B51] Mörstedt A. B. (2016). Erwartungen der Generation Z an die Unternehmen [Expectations of Generation Z Towards the Organizations]. Retrieved from: https://www.pfh.de/fileadmin/Content/PDF/forschungspapiere/vortrag-generation-z-moerstedt-ihk-goettingen.pdf

[B52] Myers K. K. Sadaghiani K. (2010). Millennials in the workplace: a communication perspective on millennials' organizational relationships and performance. J. Bus. Psychol. 25, 225–238. 10.1007/s10869-010-9172-7 20502509 PMC2868990

[B53] Niemiec R. M. (2014). “Mindfulness-based strengths practice (MBSP) for physicians: integrating core areas to promote positive health,” in Positive Health: Flourishing Lives, Well-Being in Doctors, ed M. W. Snyder (Bloomington, IN: Balboa Press), 247–263.

[B54] O'Leary K. J. Sehgal N. L. Terrell G. Williams M. V. (2012). Interdisciplinary teamwork in hospitals: a review and practical recommendations for improvement. J. Hosp. Med. 7, 48–54. 10.1002/jhm.970 22042511

[B55] Park N. Peterson C. (2009). Character strengths: research and practice. J. Coll. Charact. 10:1042. 10.2202/1940-1639.1042

[B56] Peterson C. Park N. (2006). Character strengths in organizations. J. Organ. Behav. 27, 1149–1154. 10.1002/job.398

[B57] Peterson C. Park N. Seligman M. E. P. (2005). “Assessment of character strengths,” in Psychologists' Desk Reference, 2nd Edn., eds G. P. Koocher, J. C. Norcross, and S. S. Hill (Oxford; New York, NY: Oxford University Press), 93–98.

[B58] Peterson C. Seligman M. E. P. (2004). Character Strengths and Virtues: A Handbook and Classification. Washington, DC; New York, NY: American Psychological Association.

[B59] Porter M. E. Teisberg E. O. (2007). How physicians can change the future of health care. JAMA 297, 1103–1111. 10.1001/jama.297.10.1103 17356031

[B60] Restauri N. Nyberg E. Clark T. (2019). Cultivating meaningful work in healthcare: a paradigm and practice. Curr. Probl. Diagnost. Radiol. 48, 193–195. 10.1067/j.cpradiol.2018.12.002 30638757

[B61] Schaufeli W. B. Bakker A. B. (2003). Test Manual for the Utrecht Work Engagement Scale. Unpublished manuscript. Utrecht University. Available online at: http://www.schaufeli.com (accessed January 11, 2018).

[B62] Schaufeli W. B. Bakker A. B. Salanova M. (2006). The measurement of work engagement with a short questionnaire: a cross-national study. Educ. Psychol. Meas. 66, 701–716. 10.1177/0013164405282471

[B63] Schaufeli W. B. Martínez I. M. Pinto A. M. Salanova M. Bakker A. B. (2002). Burnout and engagement in university students: a cross-national study. J. Cross-Cult. Psychol. 33, 464–481. 10.1177/0022022102033005003

[B64] Seligman M. E. P. (2002). Authentic Happiness: Using the New Positive Psychology to Realize Your Potential for Lasting Fulfillment. New York, NY: The Free Press.

[B65] Seligman M. E. P. (2011). Flourish: A Visionary New Understanding of Happiness and Well-Being. Flourish: A Visionary New Understanding of Happiness and Well-Being. New York, NY: Free Press.

[B66] Seligman M. E. P. Csikszentmihalyi M. (2000). Positive psychology: an introduction. Am. Psychol. 55, 5–14. 10.1037//0003-066X.55.1.5 11392865

[B67] Shanafelt T. D. Noseworthy J. H. (2017). Executive leadership and physician well-being: nine organizational strategies to promote engagement and reduce burnout. Mayo Clin. Proc. 92, 129–146. 10.1016/j.mayocp.2016.10.004 27871627

[B68] Slavin S. J. Schindler D. Chibnall J. T. Fendell G. Shoss M. (2012). PERMA: a model for institutional leadership and culture change. Acad. Med. 87:1481. 10.1097/ACM.0b013e31826c525a 23111270

[B69] Sonnentag S. (2003). Recovery, work engagement, and proactive behavior: a new look at the interface between nonwork and work. J. Appl. Psychol. 88, 518–528. 10.1037/0021-9010.88.3.518 12814299

[B70] Stewart M. T. Reed S. Resse J. Galligan M. M. Mahan J. D. (2019). Conceptual models for understanding physician burnout, professional fulfillment, and well-being. Curr. Probl. Pediatr. Adolesc. Health Care 49, 1–10. 10.1016/j.cppeds.2019.100658 31629639

[B71] Strecker C. Huber A. Höge T. Hausler M. Höfer S. (2019). Identifying *thriving* workplaces in hospitals: work characteristics and the applicability of character strengths at work. Appl. Res. Qual. Life 15, 437–461. 10.1007/s11482-018-9693-1 32457815 PMC7250643

[B72] Su R. Tay L. Diener E. (2014). The development and validation of the Comprehensive Inventory of Thriving (CIT) and the Brief Inventory of Thriving (BIT). Appl. Psychol. 6, 251–279. 10.1111/aphw.12027 24919454

[B73] The Wellbeing Lab (2021). Available online at: https://permahsurvey.com/the-science (accessed April 23, 2021).

[B74] Thiel M. (2015). Das Aufnahmeverfahre nan der MedUni Innsbruck [The Admission Procedure at the Medical University Innsbruck]. Available online at: https://www.thieme.de/viamedici/mein-studienort-innsbruck-1595/a/aufnahmeverfahren-meduni-innsbruck-21450.htm

[B75] Weigl M. Müller A. Vincent C. Angerer P. Sevdalis N. (2012). The association of workflow interruptions and hospital doctors' workload: a prospective observational study. BMJ Qual. Saf. 21, 399–407. 10.1136/bmjqs-2011-000188 22190539

[B76] Weintraub A. S. Sarosi A. Goldberg E. Waldman E. D. (2020). A cross-sectional analysis of compassion fatigue, burnout, and compassion satisfaction in pediatric hematology-oncology physicians in the United States. J. Pediatr. Hematol. 42, e50–e55. 10.1097/MPH.0000000000001548 31259831

[B77] West C. P. Huschka M. M. Novotny P. J. Sloan J. A. Kolars J. C. Habermann T. M.. (2006). Association of perceived medical errors with resident distress and empathy: a prospective longitudinal study. J. Am. Med. Assoc. 296, 1071–1078. 10.1001/jama.296.9.1071 16954486

[B78] Wurm W. Vogel K. Holl A. Ebner C. Bayer D. Mörkl S. (2016). Depression-burnout overlap in physicians. PLoS ONE 11:e0149913. 10.1371/journal.pone.0149913 26930395 PMC4773131

